# Organic Thin Films Enable Retaining the Oxidation State of Copper Catalysts during CO_2_ Electroreduction

**DOI:** 10.1021/acsami.3c14554

**Published:** 2024-01-26

**Authors:** Yujie Peng, Chao Zhan, Hyo Sang Jeon, Wiebke Frandsen, Beatriz Roldan Cuenya, Christopher S. Kley

**Affiliations:** † Helmholtz Young Investigator Group Nanoscale Operando CO_2_ Photo-Electrocatalysis, Helmholtz-Zentrum Berlin für Materialien und Energie GmbH, 14109 Berlin, Germany; ‡ Department of Interface Science, Fritz Haber Institute of the Max Planck Society, 14195 Berlin, Germany

**Keywords:** CO_2_ electroreduction, organic−metal interface, Cu^+^ species, electrolyte design, synergistic effect

## Abstract

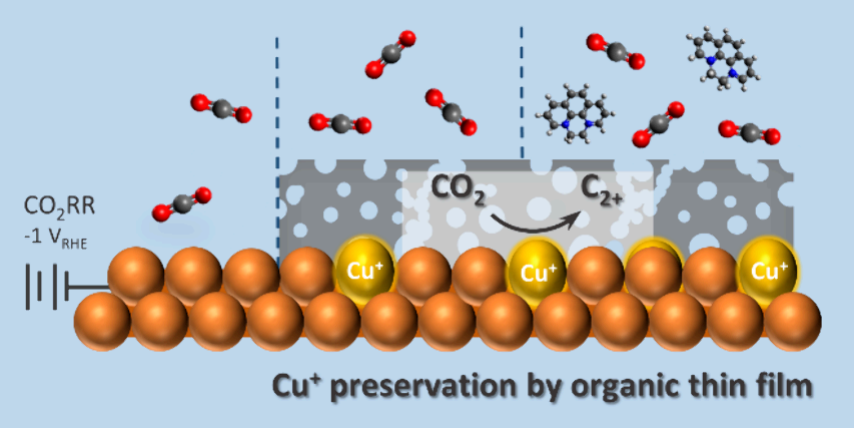

A key challenge in electrocatalysis remains controlling a catalyst’s structural, chemical, and electrical properties under reaction conditions. While organic coatings showed promise for enhancing the selectivity and stability of catalysts for CO_2_ electroreduction (CO_2_RR), their impact on the chemical state of underlying metal electrodes has remained unclear. In this study, we show that organic thin films on polycrystalline copper (Cu) enable retaining Cu^+^ species at reducing potentials down to −1.0 V vs RHE, as evidenced by *operando* Raman and *quasi in situ* X-ray photoelectron spectroscopy. *In situ* electrochemical atomic force microscopy revealed the integrity of the porous organic film and nearly unaltered Cu electrode morphology. While the pristine thin film enhances the CO_2_-to-ethylene conversion, the addition of organic modifiers into electrolytes gives rise to improved CO_2_RR performance stability. Our findings showcase hybrid metal–organic systems as a versatile approach to control, beyond morphology and local environment, the oxidation states of catalysts and energy conversion materials.

## 1 Introduction

Key catalytic reactions driven by transition metals, including the electrocatalytic reduction of CO_2_ (CO_2_RR) into chemical feedstocks or the nitrate reduction reaction (NO_3_RR) for ammonia synthesis, have been challenged by poor performance stability and low product selectivity.^1,2^ Recent *operando* spectroscopy and microscopy works have shown that the structure, oxidation state, and composition of state-of-the-art materials, particularly copper (Cu), undergo significant changes during electrocatalysis.^3−8^ Hence, alongside synthesizing catalysts featuring well-defined properties prior to reactions (“catalyst precursors”), developing strategies for controlling a catalyst’s properties during reaction (“active catalyst”) is of key importance.

Steering the oxidation state of Cu catalysts at work is of particular interest since both theoretical and experimental works have highlighted the potential of Cu^0^/Cu^+^ interfaces to promote CO_2_RR selectivity toward multicarbon (C_2+_) products.^8−12^ So far, efforts have been directed toward tuning the oxidation state of Cu electrocatalysts via pulse potentials,^13,14^ local confinement,^15^ elemental doping,^16,17^ or engineering interfaces featuring a second oxide material.^18,19^ The synergistic coexistence of Cu^+^ species and metallic Cu was reported to strengthen the *CO adsorption and inherently facilitate the carbon–carbon bond formation.^11,12^ In this context, metal–organic hybrid approaches have shown promise in influencing the oxidation state of Cu. For instance, Cu^+^ species were observed during CO_2_RR for thiol-modified Cu electrodes, which was related to the cleavage of Cu–S bonds.^20^ However, such self-assembled monolayers are typically susceptible to degradation at CO_2_RR relevant potentials and promotional effects fade rapidly.^20,21^

Recently, organic polymeric coatings received increasing attention as potential means to enhance C_2+_ selectivity and CO_2_RR performance stability.^22,23^ Specifically, promotional effects of polymer coatings on metal electrodes were rationalized by altered morphology,^23^ proton diffusion,^24^ electrode wettability,^25,26^ local pH,^27^ and stability of reaction intermediates.^28,29^ However, the impact of organic thin films on the chemical state of the underlying metal support remains unclear and unexplored. Moreover, fundamentally understanding the origin of observed promotional effects in such hybrid systems remains challenging due to the coexistence of organic coatings and organic modifiers dissolved in the electrolyte. In addition, *ex situ* microscopic characterization performed so far on hybrid systems do not allow evaluating the integrity of organic coatings and the electrode morphology during CO_2_RR. Overall, advancing rational design concepts for metal–organic CO_2_RR electrocatalysts featuring enhanced stability and selectivity necessitates (i) *in situ* real-space information on the morphology of hybrid interfaces under relevant reaction conditions, (ii) deconvolution of the effects of organic modifiers adsorbed on electrodes from those dissolved in the electrolyte, and (iii) knowledge of the impact of organic overlayers on the oxidation state of the underlying electrocatalysts during CO_2_RR.

In this work, we show that organic thin films enable preserving oxidized species of Cu electrodes during CO_2_RR. For polycrystalline Cu electrodes prefunctionalized by a prototypical phenanthrolinium-based organic thin film, *quasi in situ* X-ray photoelectron spectroscopy (XPS) and *operando* Raman spectroscopy reveal the retention of Cu^+^ species at cathodic potential down to −1.0 V vs RHE. *In situ* electrochemical atomic force microscopy (EC-AFM) uncovers the intactness of the porous organic film and the nearly unaltered morphology of the underlying Cu electrode. An increase in CO_2_-to-ethylene conversion efficiency is observed after electrode prefunctionalization, which is attributed to abundant Cu^0^/Cu^+^ interfaces and altered microenvironment of the Cu electrode, rather than changes in the electrode morphology and wettability. Potentiostatic electrochemical impedance spectroscopy (PEIS) indicates a complex interfacial structure involving the Cu electrode, organic layer, and dissolved modifiers. A synergistic effect between the electrodeposited thin film and organic modifiers in aqueous bicarbonate electrolyte is revealed and correlated with the improved CO_2_RR selectivity and stability over 10 h operation. Our findings demonstrate a route toward controlling *in situ* the oxidation state of electrocatalysts while maintaining their morphology.

## 2 Results and Discussion

**Figure 1:**
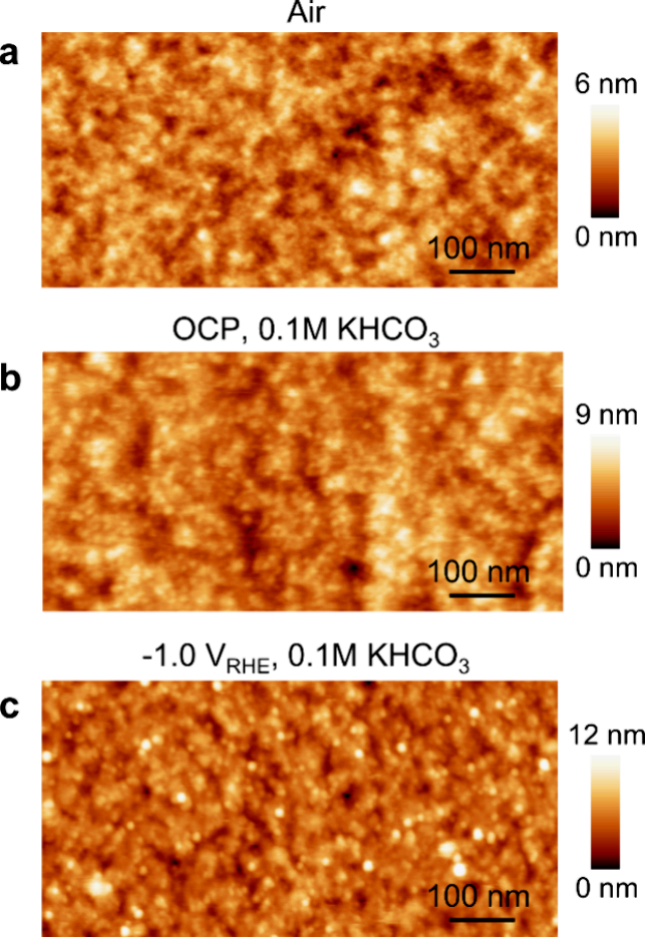
*In situ* morphology characterization of the layer-Cu. EC-AFM images of layer-Cu obtained (a) in air, (b) at OCP, and (c) at −1.0 V_RHE_ after 1 h of CO_2_RR in 0.1 M KHCO_3_.

To investigate the impact of organic thin films on the oxidation state of underlying metal electrodes during CO_2_RR, a prototypical organic–metal interface was prepared by electrodepositing *N*,*N*′-ethylene-phenanthrolinium dibromide (1-Br_2_), a previously reported precursor,^23,24^ on a polycrystalline Cu foil (denoted as layer-Cu; details on ligand synthesis and thin film preparation in Supporting Information S1 and S2). As illustrated in Figure 1a, *ex situ* AFM shows that the originally smooth electropolished Cu surface (Figure S3a) gets covered by a coarse, porous film of ∼12 nm thickness (Figure S3b,c). To evaluate the morphology of the organic thin film and potential reconstruction of the underlying Cu electrode during CO_2_RR, we perform *in situ* electrochemical atomic force microscopy. At open circuit potential (OCP), granular features of the porous organic layer are resolved in 0.1 M KHCO_3_ (Figure 1b). Figure 1c shows the organic film measured *in situ* at the highly gas-evolving potential of −1.0 V_RHE_ after 1 h CO_2_RR, indicating the high structural stability of the organic film. In contrast to a previous work,^23^ no reconstruction of the underlying Cu electrode becomes visible in our EC-AFM measurements during CO_2_RR, which can be rationalized by the different sample preparation methods (see Supporting Information S2). *Ex situ* SEM imaging confirms the absence of any significant morphological changes (Figure S4). Complementary contact angle measurements show a negligible impact of the organic thin film on the wettability of the Cu electrode surface (Figure S5).

**Figure 2:**
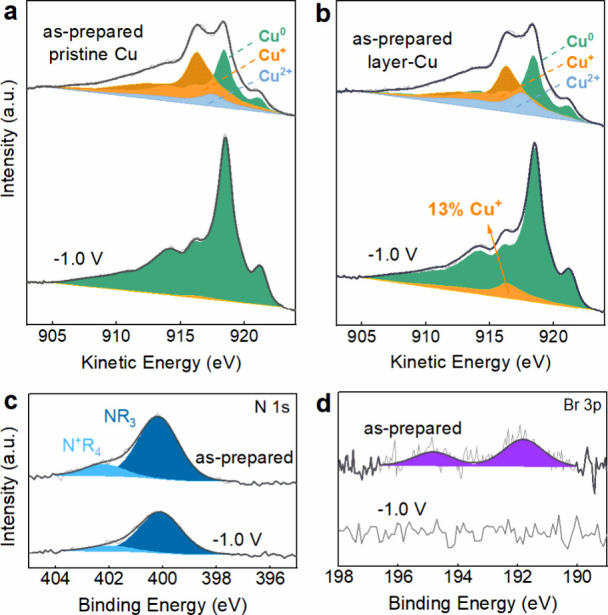
*Quasi in situ* XAES and XPS spectra of pristine Cu and layer-Cu. Cu LMM XAES spectra of (a) pristine Cu and (b) layer-Cu before and after 1 h of CO_2_RR at −1.0 V_RHE_ in 0.1 M KHCO_3_. (c) N 1s and (d) Br 3p spectra of layer-Cu before and after 1 h CO_2_RR at −1.0 V_RHE_ in 0.1 M KHCO_3_.

XPS of the pristine Cu reveals oxygen, carbon, and copper features (Figure S6), while the layer-Cu exhibits additional nitrogen and bromine signals (Figure S7 and Figure 2c,d), which are associated with the predeposited organic thin film. Quantitative elemental analysis of the as-prepared layer-Cu indicates an atomic ratio C:N of approximately 7:1 (Table S1), consistent with that of 1-Br_2_ (C_14_H_12_N_2_Br_2_), supporting the successful attachment of a 1-Br_2_-derived organic layer on the Cu surface prior to electrolysis. As shown in Figure 2a,b, X-ray Auger electron spectroscopy (XAES) indicates that both the pristine Cu and the layer-Cu samples are composed of appreciable amounts of oxide species in the initial state. Notably, *quasi in situ* XAES reveals that after 1 h of CO_2_RR at −1.0 V_RHE_, the layer-Cu sample features 13% remaining Cu^+^, whereas the unmodified Cu is nearly fully reduced. Such a clear contrast implies that the organic layer enables the retention of Cu^+^ under reducing conditions. The proportion of the quaternary (N^+^R_4_, 402.8 eV) vs tertiary (NR_3_, 400.8 eV) amine peak decreases during CO_2_RR, as N^+^R_4_ gets reduced into neutrally charged NR_3_ (Figure 2c). Notwithstanding, this reflects that the organic layer adheres to the Cu surface over the course of CO_2_RR. Figure 2d shows that a trace amount of bromide was detected on the as-prepared Cu layer sample, neutralizing the positively charged end group of the polymer, as depicted in Scheme S1. However, the initially present bromide completely leaches out of the organic thin film and diffuses into the electrolyte after 1 h of CO_2_RR at −1.0 V_RHE_, making a bromide-induced promotional effect less likely.^30,31^

**Figure 3:**
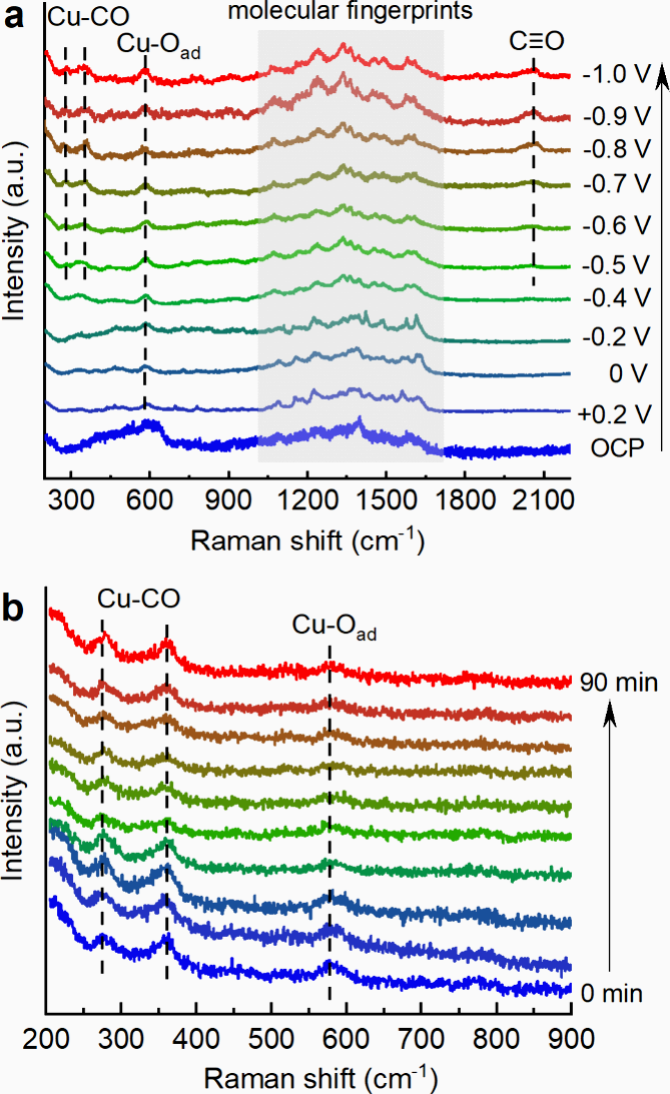
*Operando* SERS spectra of oxygen plasma-treated Cu coated by organic thin film, in CO_2_-saturated 0.1 M KHCO_3_ as a function of (a) applied potential and (b) reaction time at constant potential of −1.0 V_RHE_.

To further corroborate the effect of the organic layer on retarding the reduction of the underlying Cu oxide species during CO_2_RR, we carried out *operando* surface-enhanced Raman spectroscopy (SERS). Figure 3a depicts the SERS spectra acquired from 200 to 2200 cm^–1^ on an oxygen-plasma-roughened Cu foil covered by the organic layer in 0.1 M KHCO_3_ electrolyte as a function of the applied potential. At OCP, a broad band between ∼350 and 670 cm^–1^ is observed, which is assigned to native Cu_2_O species. Upon applying reducing potentials, the latter narrows to a peak at 585 cm^–1^, which matches the Cu–O_ad_ stretch mode.^32,33^ Importantly, the Cu–O_ad_ fingerprint persists down to strongly reducing conditions at −1.0 V_RHE_, implying that the initial oxide surface does not become fully metallic. On the contrary, the oxide-related feature diminishes already at −0.2 V_RHE_ in the absence of the organic film (Figure S8). Below −0.5 V_RHE_, CO as the key intermediate of CO_2_RR is detected (Figure 3a), with C–O stretching vibration (1970–2110 cm^–1^) as well as Cu-CO rotation (280 cm^–1^) and Cu-CO stretching (360 cm^–1^) bands.^34,35^ Moreover, the characteristic peaks of the organic film are located in the aromatic stretching region (1000–1700 cm^–1^; typically, 1612 (A_1_), 1579 (B_2_), 1487 (A_1_), 1452 (A_1_), and 1421 cm^–1^ (B_2_) belong to the phenanthroline part).^36,37^ The latter remain visible down to −1.0 V_RHE_, indicating the high stability of the organic film on Cu electrodes during CO_2_RR. Moreover, to investigate the durability of Cu oxide species, *operando* Raman was performed over an extended CO_2_RR reaction time at −1.0 V_RHE_. Notably, as shown in Figure 3b, the Cu–O_ad_ peak sustains over 90 min, validating the organic thin film’s capability of retaining oxygen on the electrode surface and stabilizing Cu^+^ species, in agreement with the *quasi in situ* XPS results.

**Figure 4:**
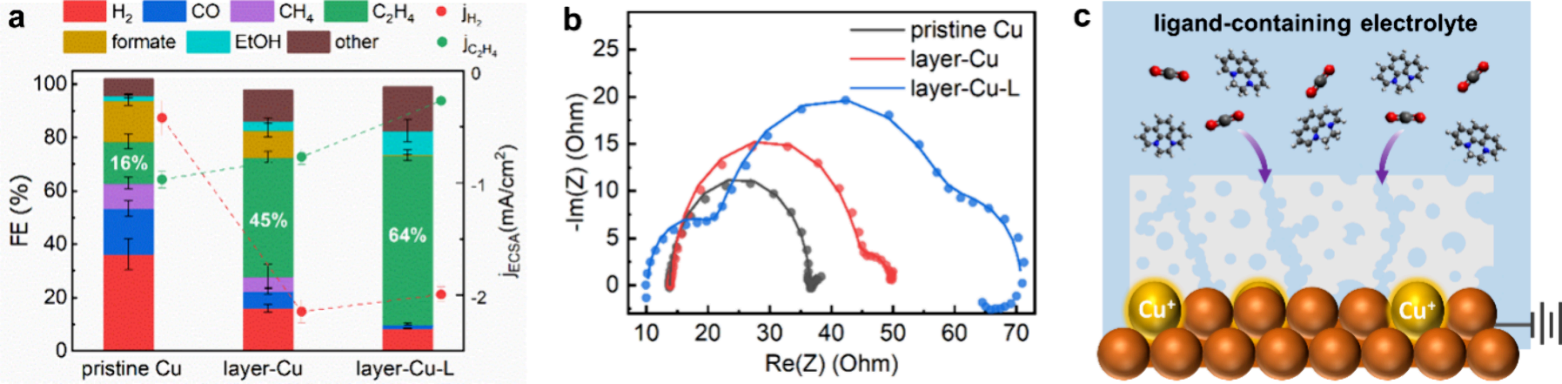
Deconvoluting the effects of electrodeposited organic thin films and dissolved organic modifiers on polycrystalline Cu-based CO_2_RR. (a) Corresponding product distribution (FE) for CO_2_RR at −1.0 V_RHE_ over 1 h and ECSA-normalized partial current densities (red and green dots depict H_2_ and C_2_H_4_, respectively) of pristine Cu and layer-Cu in 0.1 M KHCO_3_, and layer-Cu in 1-Br_2_-modified electrolyte. (b) Nyquist plots acquired for the three electrocatalytic systems as indicated at −1.0 V_RHE_ (dot for raw spectra, line for simulated spectra). (c) Schematic illustration of the layer-modified Cu electrode surface in the modifier-containing electrolyte.

To elucidate how the preserved Cu oxide species impact the CO_2_RR selectivity and to disentangle the effects resulting from the organic coating versus dissolved organic modifiers, the CO_2_RR performance of pristine Cu and modified Cu was systematically evaluated (Figure 4a). In pristine 0.1 M KHCO_3_ electrolyte, the layer-Cu exhibits an enhanced ethylene selectivity of 45% in comparison to pristine Cu (16%), at the expense of hydrogen and C_1_ products. Upon adding 10 mM 1-Br_2_ into the 0.1 M KHCO_3_ electrolyte with its bulk pH remaining constant (denoted as layer-Cu-L), the ethylene selectivity of layer-Cu is further increased to 64% Faradaic efficiency. In a previous work by Thevenon et al., a similar increase in the ethylene selectivity was ascribed to morphological changes of the Cu electrode into cubic structures, induced by the highly concentrated bromide ions.^23^ However, in this work, no large-scale surface reconstruction of the thin film-coated Cu electrode is observed after reacting in either clean or 1-Br_2_-containing electrolyte (Figure S4d,e) while the CO_2_RR selectivity significantly shifts toward ethylene even in organic modifier-free electrolyte. The latter indicates that the properties of the metal–organic interface play a more crucial role for the observed selectivity change than the electrode morphology. Furthermore, we exclude the chemical effect of bromide ions from the 1-Br_2_ electrolyte modifier on the selectivity by adding equivalent 20 mM KBr (Figure S9). Control experiments in Ar flow confirm that CO_2_, rather than the organic thin film or the ligand additive in the electrolyte, is the carbon source for CO_2_RR (Figure S10). A detailed analysis of the intrinsic activity was carried out to disentangle the contributions from organic coating and dissolved organic modifiers to ethylene formation. Upon electrodeposition of the organic film, the electrochemical surface area (ECSA) of the Cu electrode decreases from 23 to 17.5 μF/cm^2^ (Figure S11), indicating that fractions of the Cu sites are shielded by electro-grafted organic moieties, which, as revealed by *quasi in situ* XPS and *operando* Raman spectroscopy, enable the preservation of Cu^+^ species. Yet, the layer-Cu electrode features a higher intrinsic CO_2_RR activity at −1.0 V_RHE_ relative to pristine Cu (Figure S12). Specifically, as shown in Figure 4a, a fivefold increase in ethylene partial current density is obtained while the parasitic hydrogen evolution reaction (HER) is suppressed. Upon further adding 1-Br_2_ into the electrolyte, the CO_2_RR activity toward ethylene formation remains similarly high with respect to layer-Cu in pristine electrolyte. The latter suggests that the organic thin film, which actively protects the underlying Cu^+^ species, is the major contributor to the considerable increase in the ethylene selectivity. Nevertheless, the addition of 1-Br_2_ into the electrolyte suppresses HER to a greater extent, presumably due to the inhibited proton diffusion to the electrode.^24,38^Figure S12 shows the CO_2_RR selectivity and activity of the catalytic systems at various cathodic potentials.

Next, we performed PEIS for probing how the interfacial structure varies by depositing the organic film and adding 1-Br_2_ into the 0.1 M KHCO_3_ electrolyte, respectively. The Nyquist plots of the pristine Cu, layer-Cu, and layer-Cu-L recorded at −1.0 V_RHE_ are presented in Figure 4b, together with the simulated spectra based on equivalent circuits (Figure S13). For the pristine Cu foil (Figure 4b, black curve), the single semicircle is associated with a conventional double-layer interface between the electrode and the electrolyte. Electrodepositing the organic film on the polycrystalline Cu gives rise to two distinguishable arces (Figure 4b, red curve), with the second arce at low frequency indicating a newly formed electrode–film interface. The corresponding fitting is based on the interface electric circuit II (Figure S13) which has been previously used for modeling organic polymer-coated electrodes.^39^ The fitting results (Tables S2 and S3) show that the double-layer capacitance (C_dl_) of layer-Cu drops by 26% relative to that of pristine Cu, in accordance with the observed decreased ECSA calculated from cyclic voltammograms. Moreover, the capacitance deconvoluted from the electrode/organic film interface (6 mF/cm^2^) is much higher than that of the electrode/electrolyte interface (∼20 μF/cm^2^), indicating a pronounced surface area of the porous organic film.

Upon dissolving 1-Br_2_ into the electrolyte, three distinct semicircles appear (Figure 4b, blue curve), with the corresponding equivalent circuit shown in Figure S13. The third semicircle originates from the interface between the thin film and dissolved organic modifiers, which is accompanied by a negative shift of the CO_2_RR onset potential from −0.5 to −0.85 V_RHE_ (Figure S14)^40^ While the organic film is impermeable to the soluble 1-Br_2_ precursor and the growth process of the organic film is self-limited (detailed discussion in Section S2), excess organic modifiers in the electrolyte stabilize the film and concurrently prolong the lifetime of the preserved Cu^+^ species (Figure S15). This is supported by enhanced CO_2_RR performance stability of up to 10 h at −1 V_RHE_ in a 1-Br_2_ modified electrolyte (Figure S16). The PEIS measurements were carried out for each scenario over a wide range of potentials (Figure S17), with the extrapolated parameters summarized in Tables S2–S4.

Figure 4c schematically illustrates the synergistic impact of the organic film and the electrolyte additive on the CO_2_RR performance of polycrystalline Cu. During the CO_2_RR, the porous organic film enables the penetration of CO_2_ molecules and the preservation of Cu^+^ species adjacent to electrochemically accessible Cu^0^ species. The resulting Cu^0^/Cu^+^ interfaces contribute to the improved CO adsorption and lowered energy barrier for C–C dimerization, as has been shown in previous works,^9,41,42^ in addition to the impacts of organic layers on the microenvironment of electrodes.^24,43,44^ Interestingly, despite the organic thin film remaining on the electrode after 3 h of operation (Figure S18), the loss in Cu^+^ on the electrode surface leads to observed catalyst degradation in the stability test (Figure S16a). These results indicate that the Cu^+^ moieties preserved by the organic layer plays a more decisive role than the altered microenvironment (proton/CO_2_ mass transport, confinement of key intermediates) for the enhanced C_2+_ selectivity. While, thermodynamically, solely metallic Cu species are expected at considered potentials down to −1.0 V_RHE_, the observation of mixtures of Cu^0^/Cu^+^ species is rationalized by altered kinetics on coated Cu surface areas and dynamic CO_2_RR environment, such as local pH^45^ and subsurface oxygen.^10^ The addition of organic modifiers into the electrolyte has a minor effect on the ethylene formation rate; however, it distinctly increases both the robustness of the thin film and the CO_2_RR performance stability.

## 3 Conclusions

In summary, we systematically deconvoluted how organic thin films electrodeposited on polycrystalline Cu and organic modifiers dissolved in aqueous electrolyte impact CO_2_RR. By stepwise decorating preoxidized Cu electrode surfaces and tailoring the electrolyte composition, we observed progressively increased CO_2_-to-ethylene selectivity. We unraveled the capability of the porous organic thin films to preserve Cu^+^ species throughout extended periods of the CO_2_RR at potentials as low as −1.0 V_RHE_, contributing to promoted C–C coupling. Moreover, organic additives in aqueous bicarbonate electrolyte were shown to distinctly increase the stability of the CO_2_RR performance. *In situ* EC-AFM conducted during highly gas-evolving CO_2_RR validated the integrity of the hybrid metal–organic interfaces and was shown to be a powerful tool for resolving the morphology of delicate organic films under relevant reaction conditions.

As highlighted in our study, building reliable structure–property relationships for hybrid metal–organic electrocatalysts requires considering the impacts of organic overlayers not only on the electrolyte microenvironment (e.g., pH, diffusion) but also on the chemical properties of the underlying electrode. Promising avenues include employing metal–organic approaches to concomitantly control the oxidation state, morphology, and microenvironment of materials for various catalytic reactions and protecting bulk/nanoparticle electrodes from degradation through organic coatings and electrolyte modification. This will advance strategies for effectively regulating electrochemical processes in catalysis as well as energy conversion and storage.

## 4 Experimental Methods

### 4.1 Organic Ligand Synthesis

Following a previously reported synthetic protocol,^23^ 1.5 g of 1,10-phenanthronline (Merck, ≥99%) and 15 mL of dibromoethane (Sigma-Aldrich, ≥98%) were added in a round flask and stirred for 18 h at 110 °C. After washing with hexane and acetone each for three times and centrifuging at 9000 rpm for 10 min, the product was obtained and then dried in an oven at 50 °C.

### 4.2 Electrode Preparation

Commercial polycrystalline Cu foil (Alfa Aesar, 99.9999%) was rinsed with EtOH to remove organic impurity, followed by flushing with a large amount of deionized water (Purelab, resistivity = 18.2 MΩ). The Cu foil was electropolished by applying +4 V vs Pt in phosphoric acid (ITW reagent, 85 wt %) for 4 min, and then cleaned thoroughly with deionized water, and blow-dried with nitrogen gas. The layer-modified Cu (L-Cu) was prepared by dipping the electropolished Cu foil in 10 mM 1-Br_2_ containing 0.1 M KHCO_3_ solution, subsequently performing five linear sweeps from 0 V to −1.0 V_RHE_ at a scan rate of 20 mV/s.

### 4.3 CO_2_RR Measurements

The electrocatalytic performance of the catalyst was evaluated by conducting chronoamperometry (CA) measurement for 1 h in a gastight H-type cell. The anode and cathode compartments were separated by an anion exchange membrane (Selemion AMV, AGC Inc.), and each of them was filled with 20 mL of electrolyte. 0.1 M KHCO_3_ electrolyte was prepared by constantly feeding CO_2_ gas (99.995%) into 0.05 M K_2_CO_3_ (Alfa Aesar, 99.997%). The electrolyte was presaturated with CO_2_ prior to the measurements, and CO_2_ gas was constantly purged at a flow rate of 20 sccm during the measurement. Platinum mesh (MaTecK, 3600 mesh cm^–2^) and a reversible hydrogen electrode (RHE, HydroFlex Gakatel) were used as counter and reference electrodes, respectively. 85% of the Ohmic drop was corrected during the electrolysis, the remaining 15% was postcorrected.

### 4.4 Product Analysis

The gas products (hydrogen, carbon monoxide, methane, ethylene) produced in the cathodic compartment were detected by an online gas chromatograph (GC, Agilent 8860) equipped with a thermal conductivity detector (TCD) and a flame ionization detector (FID). The liquid products were measured depending on the electrolyte composition. For ligand-free electrolyte, formate was analyzed with high-performance liquid chromatography (HPLC, Shimadzu Prominence) and alcohols and aldehydes were quantified using a liquid GC (Shimadzu 2010 Plus). For ligand-containing electrolyte, the liquid products were analyzed by ^1^H NMR (Bruker, 600 MHz) with the water suppression technique and DMSO as internal standard.

### 4.5 Electrochemical Atomic Force Microscopy

EC-AFM images were obtained in amplitude modulation mode using a commercial atomic force microscope (Cypher VRS1250, Asylum Research/Oxford Instruments). High-frequency cantilevers (Arrow UHF Au, NanoWorld AG) were cleaned with mild argon plasma prior to use. The EC cell consists of a platinum ring as counter electrode and a silver wire as pseudo reference electrode. A potentiostat (BioLogic, SP-300) was used for regulating the applied potential. The AFM images were analyzed by using WSxM software.

### 4.6 Contact Angle Measurement

Contact angles were measured with an OCA 50 (Data Physics Instruments) by putting a volume-controlled 0.1 M KHCO_3_ droplet on freshly prepared samples. The contact angle values were calculated automatically using data physics software.

### 4.7 *Quasi In Situ* X-ray Photoelectron Spectroscopy

*Quasi in situ* XPS experiments were performed in a custom-made electrochemical cell directly attached to the ultrahigh vacuum (UHV) system. After the electrochemical treatment, the sample was transferred to the XPS chamber under Ar flow without air exposure. The electrochemical measurements were carried out with a potentiostat (Autolab PGSTAT302N); a platinum mesh and leak-free Ag/AgCl were used as the counter and reference electrodes, respectively. XPS measurements were performed with a commercial Phoibos 100 analyzer (SPECS GmbH, Epass = 20 eV) and an XR50 (SPECS GmbH) X-ray source with an Al Kα source (*E*_Kα_ = 1486.7 eV). Spectra were processed with Casa XPS software. Cu 2p_3/2_ peaks were calibrated with the standard value of 932.67 eV, and the calibration was then propagated to the other spectra.

### 4.8 *Operando* Surface-Enhanced Raman Spectroscopy

Polycrystalline Cu foil was treated with oxygen plasma (15 W, 400 Torr, 2 min), and the organic layer was electrodeposited following the same procedure described before. Subsequently, the sample was cleaned with deionized water and dried with nitrogen. The *operando* SERS spectra were recorded by means of a Renishaw (InVia Reflex) confocal Raman microscope with a 785 nm laser. A water immersion objective with a long working distance (Leica Microsystems, 63× , 0.9 numerical aperture) covered by a Teflon film (DuPont, film thickness of 0.013 mm) was used for the operando measurements in the electrolyte. The electrochemical measurements were performed in a home-built Teflon electrochemical cell, equipped with a leak-free Ag/AgCl electrode as the reference electrode and a Pt wire as the counter electrode. Typically, 15 mL of CO_2_-saturated 0.1 M KHCO_3_ electrolyte was added, continuously purged with CO_2_ during the experiment. For the potential-dependent experiment, each potential was applied for at least 10 min before collecting the spectra to ensure a steady state is achieved. Background subtraction was performed for all spectra, which were subsequently normalized by the spectrum acquired at −0.4 V_RHE_. Spectra at −0.8, −0.9, and −1.0 V_RHE_ are scaled by two times and smoothed for clarity by averaging three adjacent data points.

### 4.9 Potentiostatic Electrochemical Impedance Spectroscopy

PEIS measurements were conducted at different operating potentials with varied frequencies ranging from 100 kHz to 80 mHz. All of the electrochemical measurements were performed using a BioLogic SP-300 workstation. The Nyquist plots were fitted with equivalent circuits by using the EC-Lab software.

## Supporting Information Available

Synthesis of the 1-Br2 organic modifier; electrodeposition of the organic thin film on Cu electrodes; *ex situ* AFM characterization: thickness of the as-prepared organic layer; *ex situ* SEM characterization; contact angle measurements of pristine Cu and layer-Cu; *quasi in situ* XPS spectra of pristine Cu before and after CO_2_RR at −1.0 V_RHE_ in 0.1 M KHCO_3_; *quasi in situ* XPS spectra of layer-Cu before and after CO_2_RR at −1.0 V_RHE_ in 0.1 M KHCO_3_; *operando* Raman spectra of pristine oxygen plasma treated copper foil; disentangling the effects of bromide anions and phenanthrolinium cations from 1-Br_2_ electrolyte modifier on CO_2_RR performance; determination of the carbon source for CO_2_RR; electrochemical surface areas of pristine Cu and layer-Cu; CO_2_RR performance of examined catalytic systems in a wide potential range; equivalent circuits for fitting potentiostatic electrochemical impedance spectra; CO_2_RR onset shift in the presence of the organic modifier in the electrolyte; *quasi in situ* XPS spectra of layer-Cu after CO_2_RR at -1.0 V_RHE_ in 1-Br_2_-modified 0.1 M KHCO_3_; enhanced CO_2_RR performance stability of layer-Cu in 1-Br_2_-modified 0.1 M KHCO_3_; Nyquist plots of all examined catalytic systems at various potentials; and *quasi in situ* XPS spectra of layer-Cu after 3 h of CO_2_RR at −1.0 V_RHE_ in 0.1 M KHCO_3_ (PDF)
