# Supplementary material for: Organic Thin Films Enable Retaining the Oxidation State of Copper Catalysts during CO2 Electroreduction
Source: ACS Appl Mater Interfaces. 2024 Jan 26;16(5):6562–8. doi: 10.1021/acsami.3c14554 (PMC10859887; doi:10.1021/acsami.3c14554)
Supplement: Supplementary file 1 — am3c14554_si_001.pdf [file am3c14554_si_001.pdf]

Supporting Information

Organic Thin Films Enable Retaining the Oxidation State of Copper Catalysts during CO_2 Electroreduction

Yujie Peng,[†,‡] Chao Zhan,[‡] Hyo Sang Jeon,[‡] Wiebke Frandsen,[‡] Beatriz Roldan Cuenya,[‡] and Christopher S. Kley[*,†,‡]

[†]Helmholtz Young Investigator Group Nanoscale Operando CO_2 Photo-Electrocatalysis, Helmholtz-Zentrum Berlin für Materialien und Energie GmbH, 14109 Berlin, Germany

[‡]Department of Interface Science, Fritz Haber Institute of the Max Planck Society, 14195 Berlin, Germany

*Email: christopher.kley@helmholtz-berlin.de, kley@fhi.mpg.de

Contents

1) Synthesis of the 1-Br_2 organic modifier
2) Electrodeposition of the organic thin film on Cu electrodes
3) *Ex situ* AFM characterization: thickness of the as-prepared organic layer
4) *Ex situ* SEM characterization
5) Contact angle measurements of pristine Cu and layer-Cu
6) *Quasi in situ* XPS spectra of pristine Cu before and after CO_2RR at -1.0 V_{RHE} in 0.1 M $KHCO_3$
7) *Quasi in situ* XPS spectra of layer-Cu before and after CO_2RR at -1.0 V_{RHE} in 0.1 M $KHCO_3$
8) *Operando* Raman spectra of pristine oxygen plasma treated copper foil
9) Disentangling the effects of bromide anions and phenanthrolinium cations from 1-Br_2 electrolyte modifier on CO_2RR performance
10) Determination of the carbon source for CO_2RR
11) Electrochemical surface areas of pristine Cu and layer-Cu
12) CO_2RR performance of examined catalytic systems in a wide potential range
13) Equivalent circuits for fitting potentiostatic electrochemical impedance spectra
14) CO_2RR onset shift in the presence of the organic modifier in the electrolyte
15) *Quasi in situ* XPS spectra of layer-Cu after CO_2RR at -1.0 V_{RHE} in 1-Br_2-modified 0.1 M $KHCO_3$
16) Enhanced CO_2RR performance stability of layer-Cu in 1-Br_2-modified 0.1 M $KHCO_3$
17) Nyquist plots of all examined catalytic systems at various potentials
18) *Quasi in situ* XPS spectra of layer-Cu after 3h of CO_2RR at -1.0 V_{RHE} in 0.1 M $KHCO_3$

1) Synthesis of the 1-Br_2 organic modifier

The synthetic protocol of N, N'-ethylene-phenanthrolinium dibromide (1-Br_2) was adapted from a prior work.[1] Specifically, 1.5 g of 1,10-phenanthronline (Merck, ≥99%) and 15 mL of dibromoethane (Sigma Aldrich, ≥98%) were added in a round flask, then stirred for 18 h at 110 °C. After washing with hexane and acetone each for 3 times and centrifuging at 9000 rpm for 10 min, a yellow product was obtained and kept in oven at 50 °C to dry out the solvent. The high purity of as-sythesized 1-Br_2 modifier was confirmed by nuclear magnetic resonance spectroscopy (NMR), see Figure S1 below.

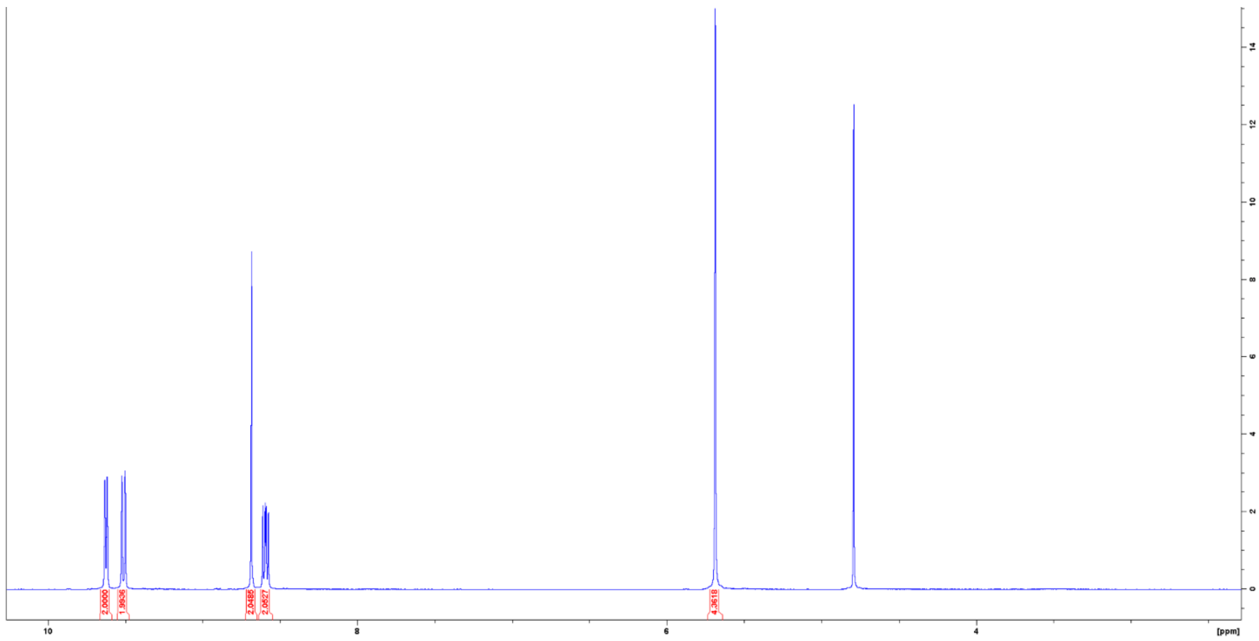

Figure S1. The ^1H NMR spectrum of as-synthesized 1-Br_2 modifier dissolved in D_2O.

2) Electrodeposition of the organic thin film on Cu electrodes

The polycrystalline Cu electrode was functionalized by performing linear sweep voltammetry in 10 mM 1-Br_2 containing bicarbonate solution. The solid red curve presents the first LSV acquired in the modifier-containing electrolyte, the broad peaks are ascribed to the layer formation, which is absent in the modifier-free electrolyte (black curve). However, the second sweep in the presence of modifiers appears to be smooth, indicating that the deposited layer is non-conductive and its growing process is self-limiting.[2] In the previous work by Peter and Agapie[1], PEIS cycles were actively-performed at OCP, inducing restructuring of the underlying Cu surface and cube-formation, prior to performing CO_2RR during which the thin film was formed *in situ*. In our work, we directly performed LSV to both accelerate the formation of the organic thin film and prevent Cu electrode reconstruction. Upon thin film preparation, the sample was rinsed with DI water and dried, prior to performing CO_2RR experiments in separate electrolyte environment.

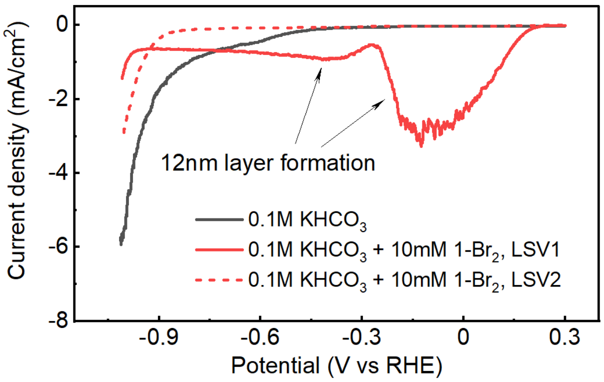

Figure S2. Electrodeposition of the organic film on the polycrystalline Cu electrode. (a) Linear sweep voltammetry recorded on a Cu foil in 0.1 M $KHCO_3$ without and with 10 mM 1-Br_2.

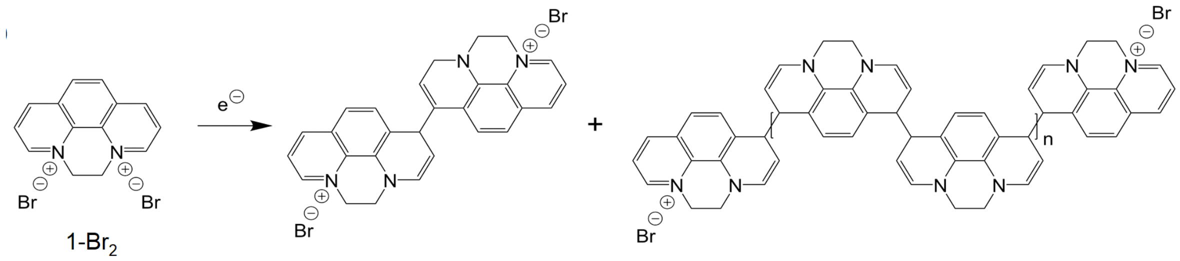

Scheme S1. The electrochemical reduction of 1-Br_2 to its dimer and oligomer.

3) *Ex situ* AFM characterization: thickness of the as-prepared layer

Figure S3a is the AFM images of a pristine Cu, featuring a lower surface roughness (RMS roughness: 0.23) than that of layer-Cu (Figure 1a, RMS roughness: 0.76). To measure the thickness of the layer, contact mode is applied for scratching the organic film in an area of 500×500 nm, then tapping mode is applied for imaging the scratched area in a larger scale of 2.0×2.0 μm, as shown in Figure S3b. Figure S3c shows the height profile of the horizontal line in Figure S3b, the flat bottom of the valley indicates the Cu substrate exposed after the scratching procedure, surrounded by piled layer residues. The as-deposited layer thickness of ca. 12 nm is determined by the height difference between the scratched and unscratched area.

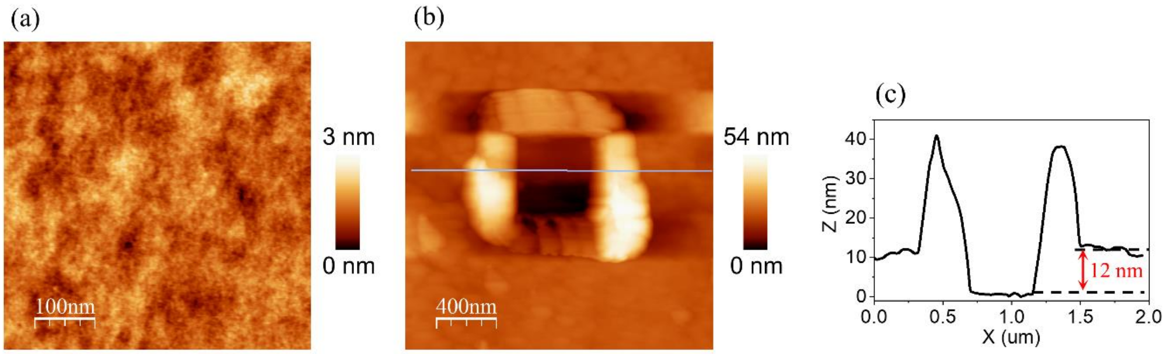

Figure S3. Morphology of pristine Cu and thickness of the as-deposited layer on Cu. *Ex situ* AFM images of (a) pristine Cu; (b) layer-Cu with the organic layer being stratched using contact mode with deflection setpoint of 2.0 V; (c) height profile of the horizontal line in (b). All the AFM images are processed with WSxM software.[3]

4) *Ex situ* SEM characterization

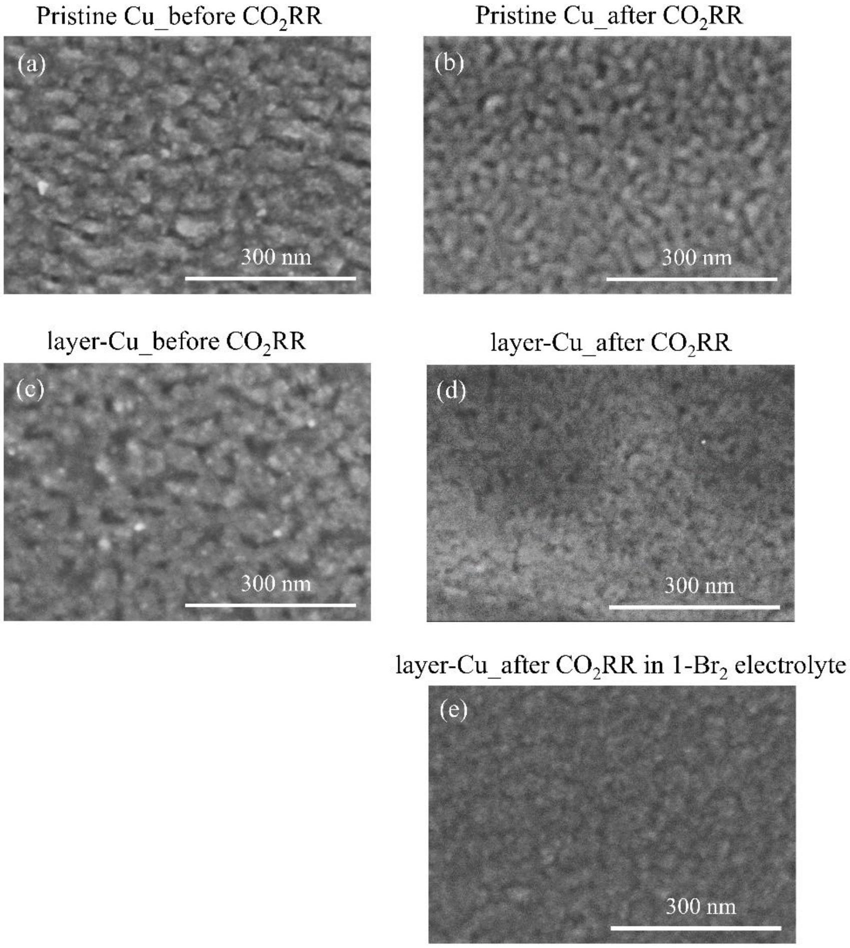

Figure S4. SEM images of (a, b) pristine Cu and (c,d) layer-Cu before and after 1 hour CO_2RR in 0.1M $KHCO_3$ at -1.0 V_{RHE}, and (e) layer-Cu after 1 hour CO_2RR in 10 mM 1-Br_2 + 0.1 M $KHCO_3$ at -1.0$V_{RHE.}$

5) Contact angle measurements of pristine Cu and layer-Cu

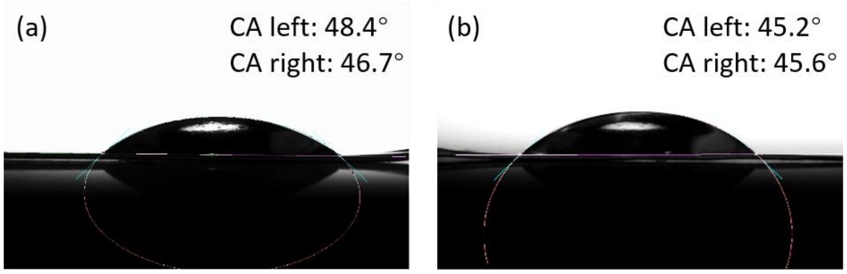

Figure S5: Contact angle measurements with a droplet of 0.1 M $KHCO_3$ aqueous electrolyte on the as-prepared (a) pristine Cu and (b) layer-Cu.

6) *Quasi in situ* XPS spectra of pristine Cu before and after CO_2RR at -1.0 V_{RHE} in 0.1M $KHCO_3$

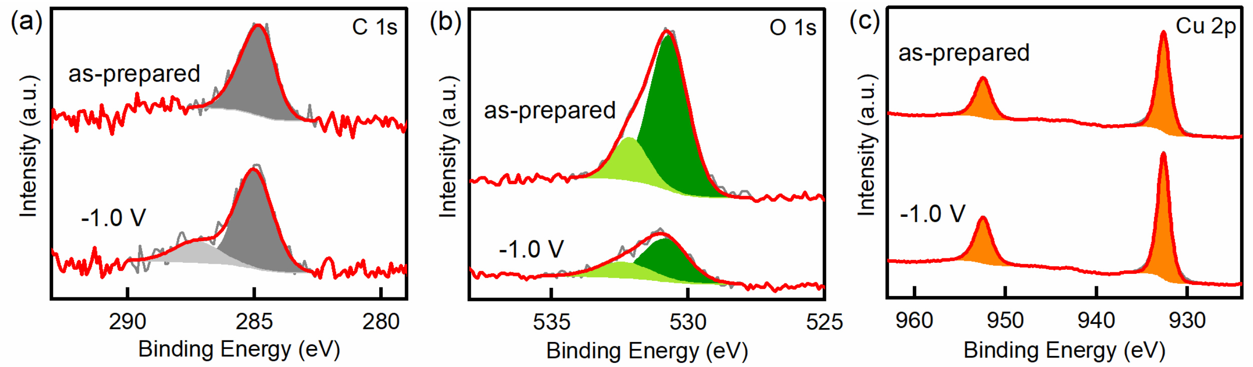

Figure S6. *Quasi in situ* XPS on pristine Cu. (a) C 1s, (b) O 1s, and (c) Cu 2p of pristine Cu before and after 1h CO_2RR at -1.0 V_{RHE} in 0.1 M $KHCO_3$.

7) *Quasi in situ* XPS spectra of layer-Cu before and after CO_2RR at -1.0 V_{RHE} in 0.1 M $KHCO_3$

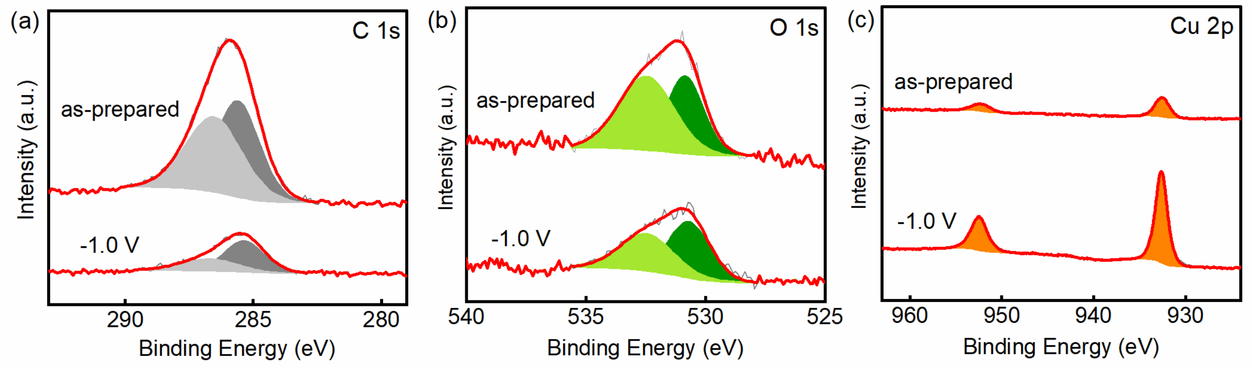

Figure S7. *Quasi in situ* XPS on layer-Cu. (a) C 1s, (b) O 1s, and (c) Cu 2p of layer-Cu before and after 1h CO_2RR at -1.0 V_{RHE} in 0.1 M $KHCO_3$. The N 1s and Br 3p of layer-Cu are shown in Figure 2c and 2d.

	Atomic ratio (%)				
	O	C	N	Cu	Br
As-prepared layer-Cu	10.1	69.9	9.1	10.7	1.2
After CO_2RR in 0.1 M $KHCO_3$	9.1	24.1	1.9	64.9	-
After CO_2RR in 0.1 M $KHCO_3$ + 10 mM 1-Br_2	6.3	75.2	10.2	6.9	1.4

Table S1. Quantitative elemental analysis of layer-Cu before and after CO_2RR at -1.0 V_{RHE} for 1h in 0.1 M $KHCO_3$ without and with 10 mM 1-Br_2.

8) *Operando* Raman spectra of pristine oxygen plasma treated copper foil

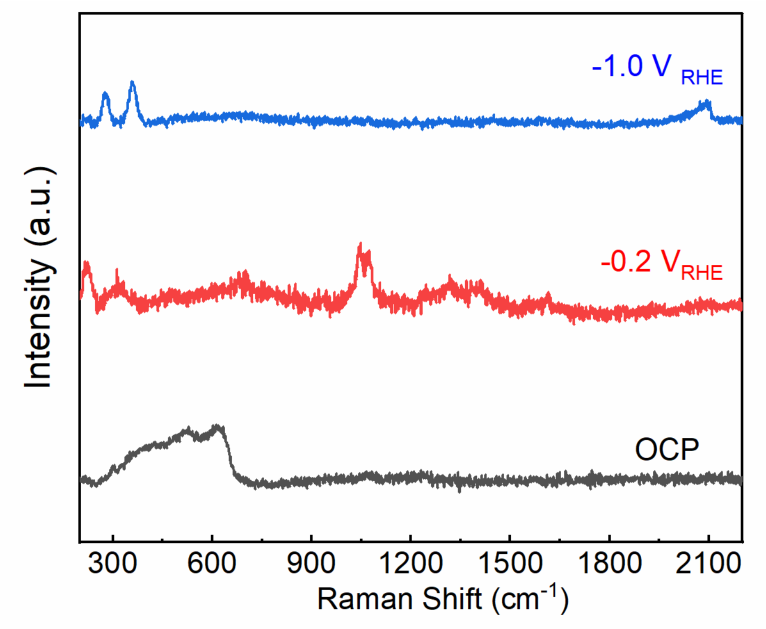

Figure S8. *Operando* Raman on pristine oxygen-plasma treated Cu foil at varied potentials in 0.1 M $KHCO_3$.

9) Disentangling the effects of bromide anions and phenanthrolinium cations from 1-Br_2 electrolyte modifier on CO_2RR performance

The ethylene selectivity obtained in 20 mM KBr-containing electrolyte is close to that of the layer-Cu in pristine electrolyte, indicating the bromide anions are not responsible for the observed selectivity enhancement to 63%, but the phenanthrolinium cations from the dissolved 1-Br_2 modifiers.

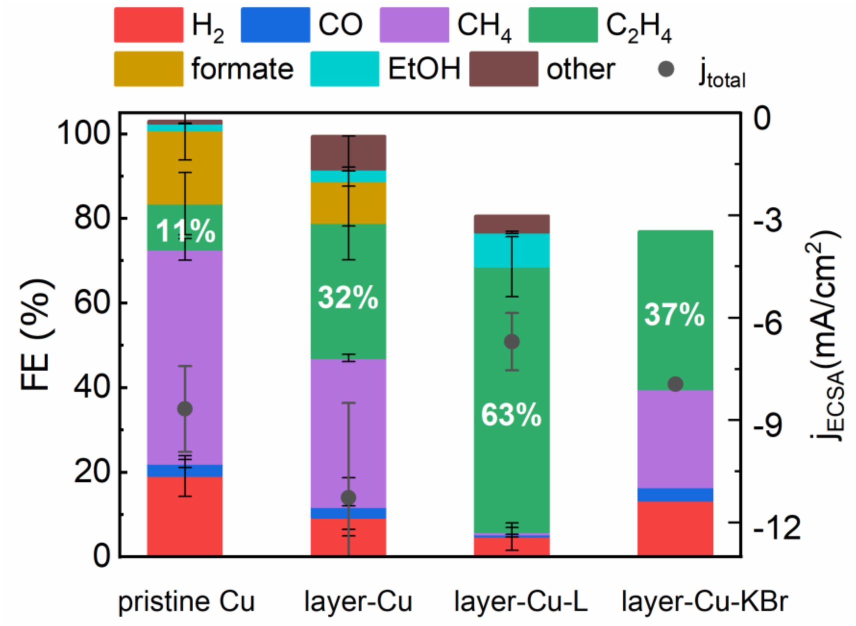

Figure S9. Selectivity and intrinsic activity of pristine Cu and layer-Cu in clean 0.1M $KHCO_3$ electrolyte, layer-Cu in 10 mM 1-Br_2 ligand-containing (layer-Cu-L) and in 20 mM KBr-containing (layer-Cu-KBr) electrolyte at -1.1V_{RHE} over 1h CO_2RR.

10) Determination of the carbon source for CO_2RR

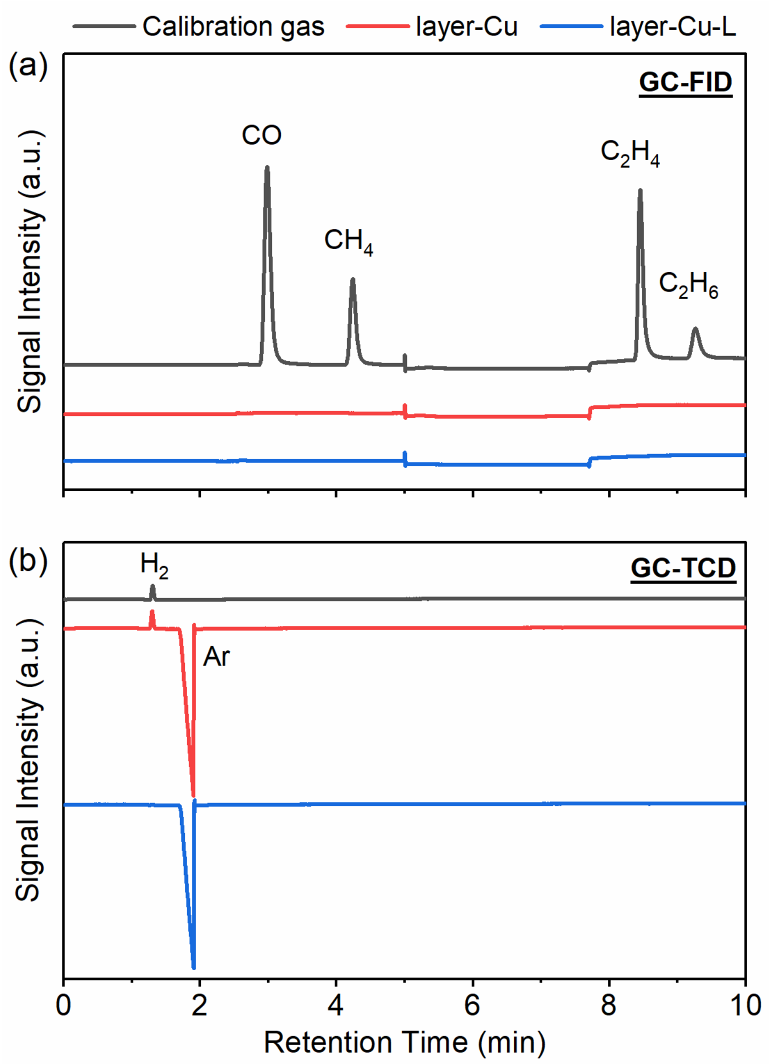

Figure S10. Gas chromatograms for determination of carbon source by online GC. (a) FID and (b) TCD signals of calibration gas, products of layer-Cu and layer-Cu-L during electrolysis in Ar atmosphere at -1.0V_{RHE}.

11) Electrochemical surface areas of pristine Cu and layer-Cu

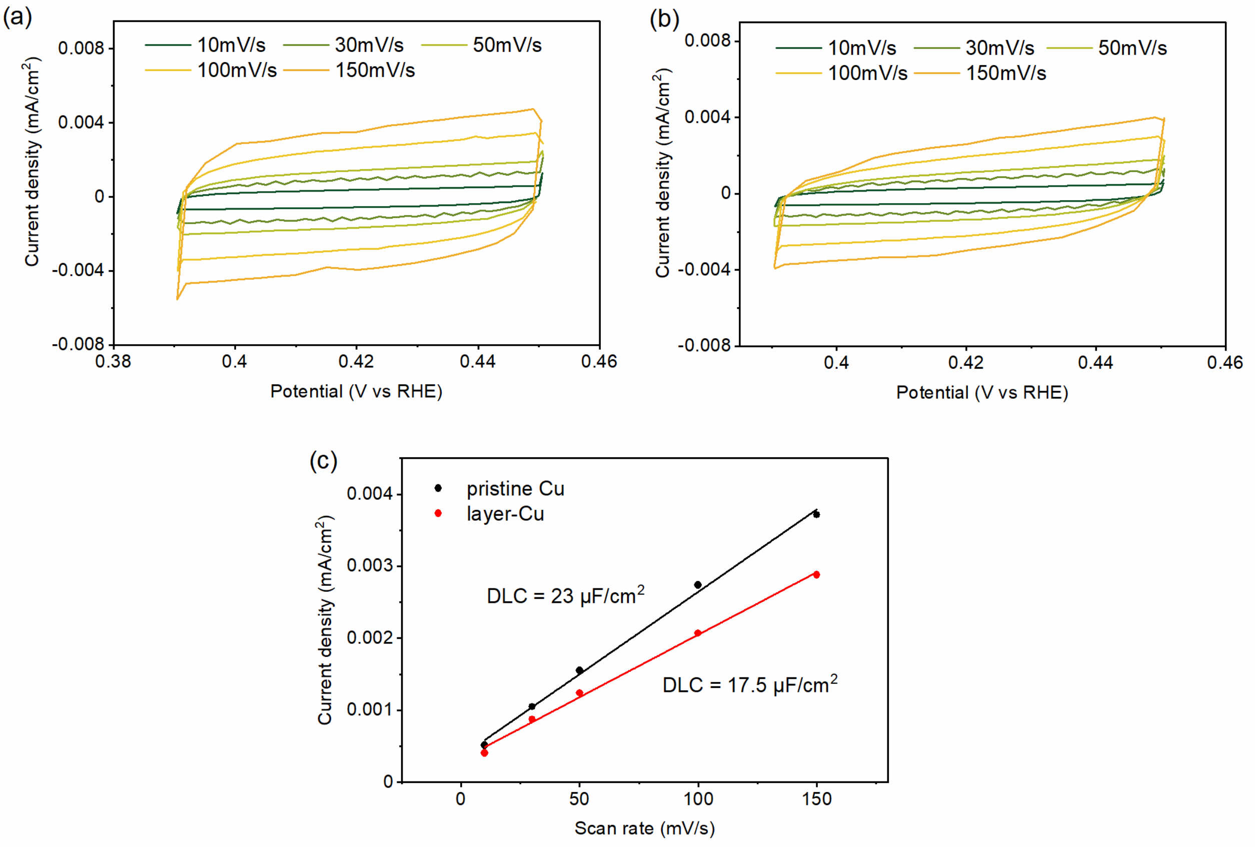

Figure S11. Electrochemical surface area measurement. Cyclic voltammetry measurements on (a) pristine Cu and (b) layer-Cu in a non-Faradaic region at varied scan rate in 0.1 M $KHCO_3$, (c) double layer capacitance of pristine Cu and layer-Cu derived from (a) and (b).

12) CO_2RR performance of examined catalytic systems in a wide potential range

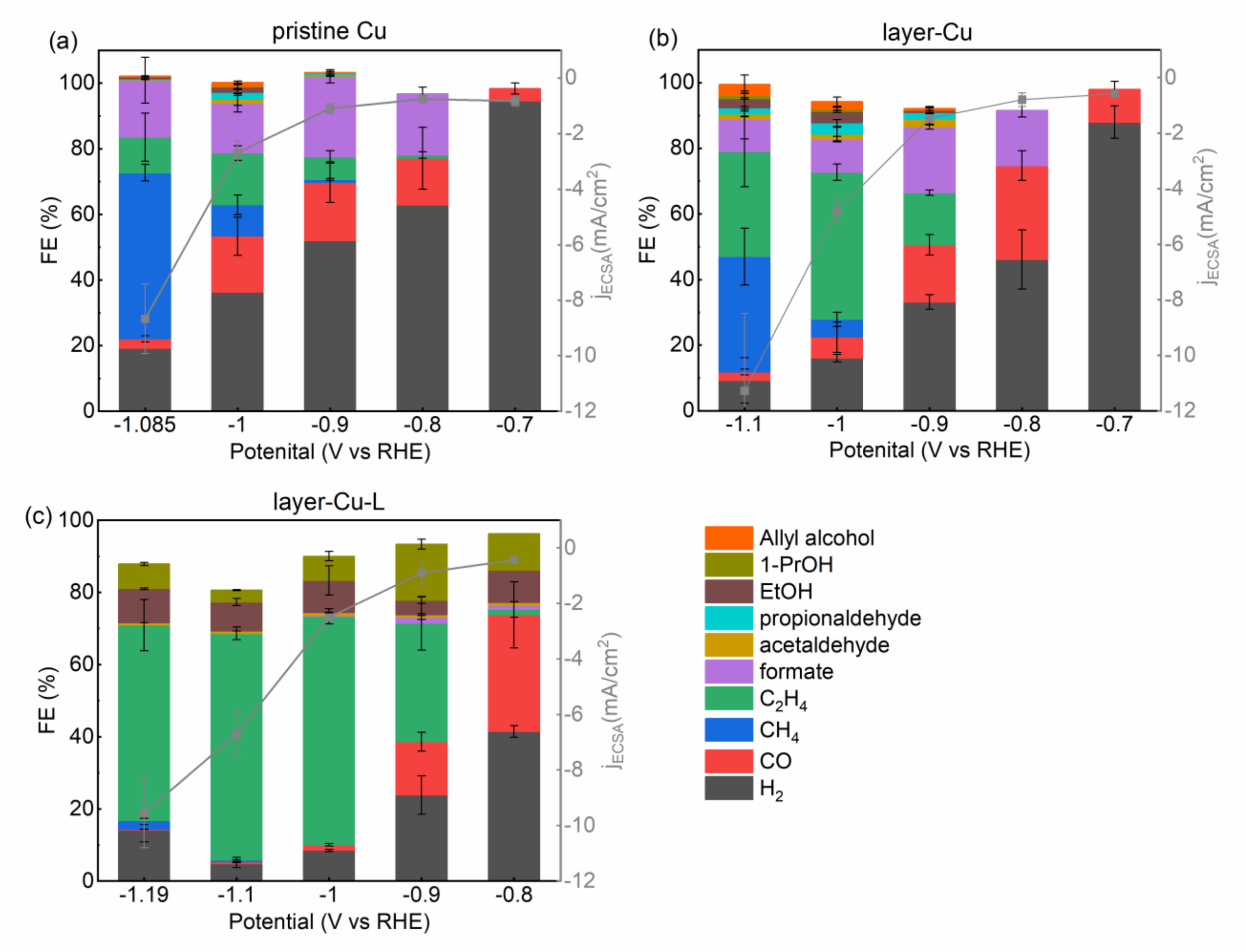

Figure S12. Selectivity and activity of (a) pristine Cu, (b) layer-Cu in pristine 0.1 M $KHCO_3$ electrolyte and (c) layer-Cu in ligand-containing 0.1 M $KHCO_3$ electrolyte in CO_2 atmosphere over 1h CO_2RR as a function of potential. Error bars are standard deviations based on at least two measurements.

13) Equivalent circuits for fitting potentiostatic electrochemical impedance spectra

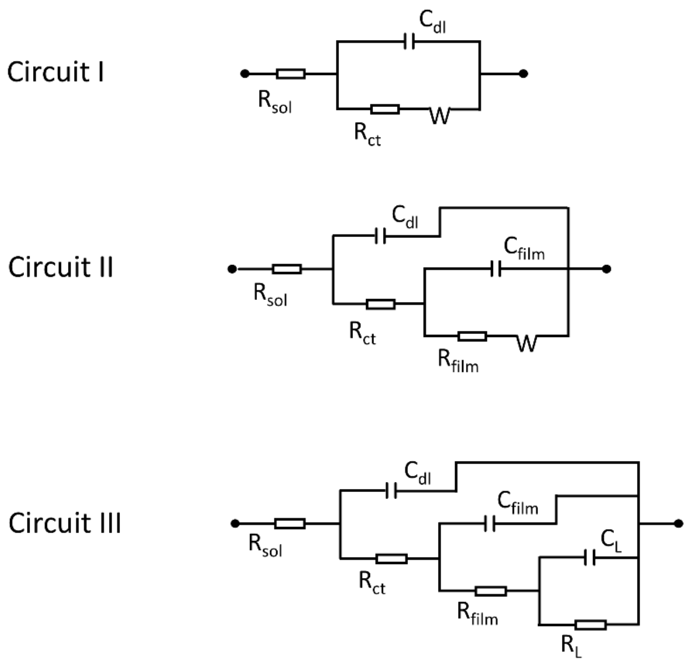

Figure S13. Equivalent circuits for simulating the Nyquist plots of pristine Cu, layer-Cu and layer-Cu-L (from top to bottom). Circuit I is known as Randle circuit for modelling planar metal electrode, where R_{sol} represents the bulk solution resistance, C_{dl} represents the double layer capacitance, R_{ct} represents the charge transfer resistance, the Warburg element is for modelling the diffusion limit. Circuit II has an extra RC element in series to R_{ct} so as to model the interface between the Cu electrode and organic film. Circuit III has one more RC element so as to model the ligand diffusion layer from the electrolyte to the organic film.

14) CO_2RR onset shift in the presence of the organic modifier in the electrolyte

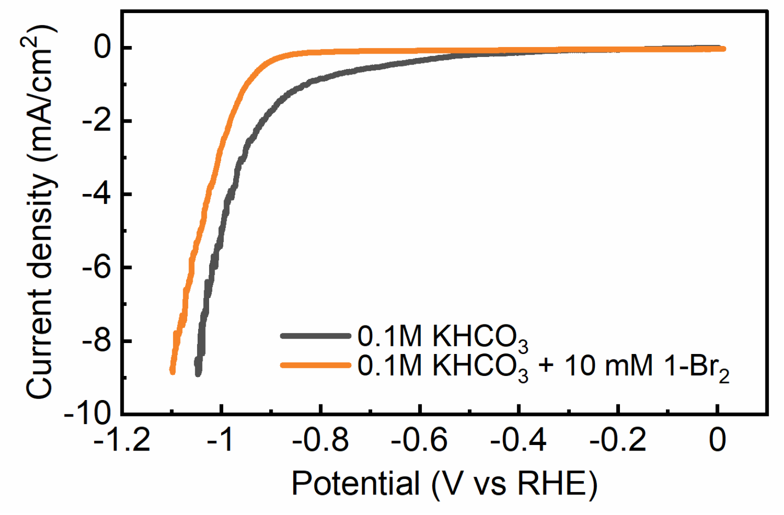

Figure S14. Linear sweep voltammetry of layer-Cu in 0.1 M $KHCO_3$ without and with 10 mM 1-Br_2.

15) *Quasi in situ XPS* spectra of layer-Cu after CO_2RR at -1.0 V_{RHE} in 1-Br_2-modified 0.1 M $KHCO_3$

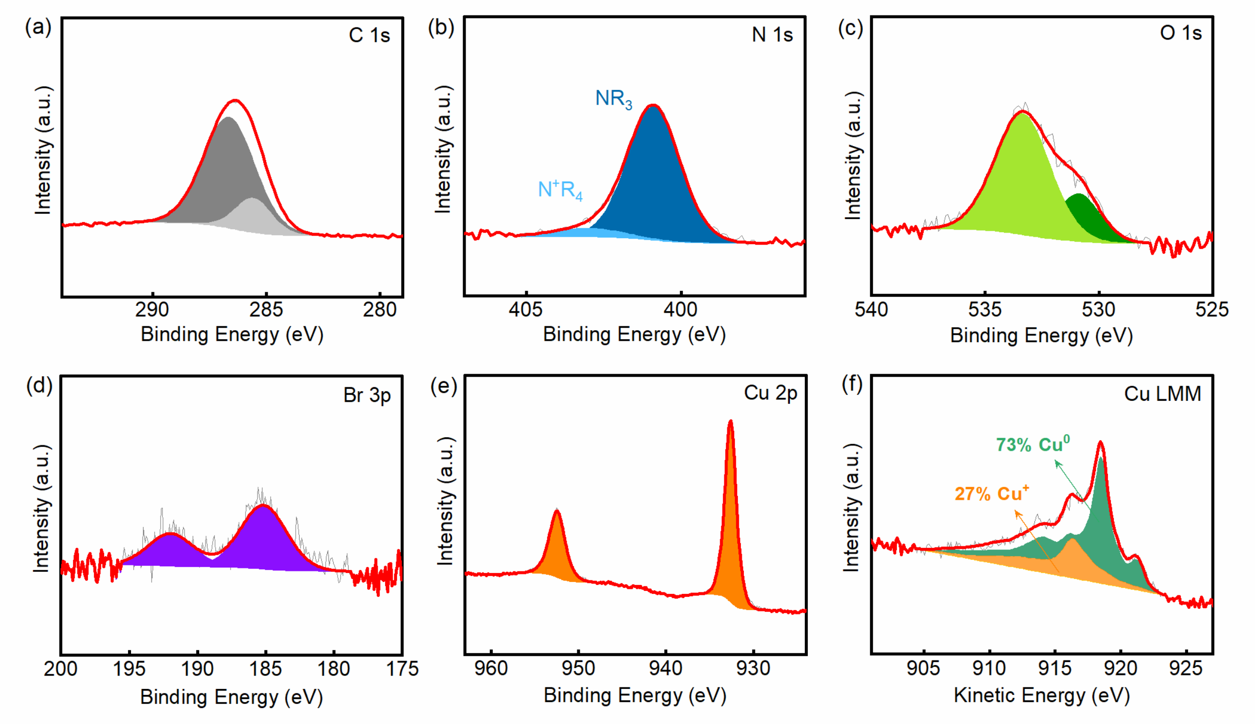

Figure S15. *Quasi in situ* XPS spectra of layer-Cu. (a) C 1s, (b) N 1s, (c) O 1s, (d) Br 3p, (e) Cu 2p, and (f) Cu LMM after 1h CO_2RR at -1.0 V_{RHE} in modifier-containing 0.1 M $KHCO_3$.

16) Enhanced CO_2RR performance stability of layer-Cu in 1-Br_2-modified 0.1 M $KHCO_3$

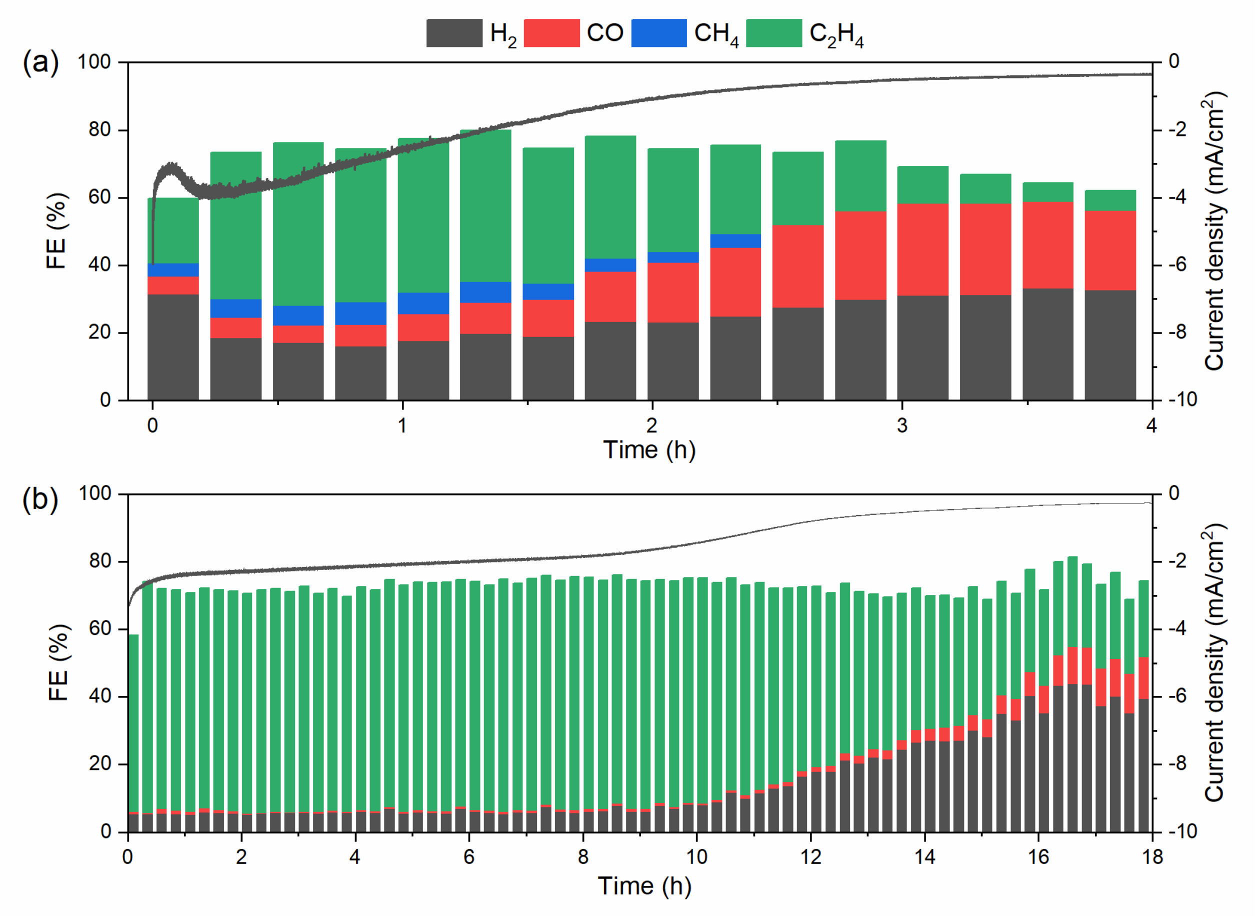

Figure S16. Selectivity and current density of layer-Cu in 0.1 M $KHCO_3$ (a) without, and (b) with 10 mM 1-Br_2 additive over extended CO_2RR reaction time.

17) Nyquist plots of all examined catalytic systems at various potentials

For each catalytic systems, PEIS was measured at varied potentials. Generally, the semicircles attenuate as the potential steps cathodically, which indicates decreased resistance. It is noted that the overall resistance decreases sharply from -0.9 V to -0.1V, consistent with the cathodically shifted onset potential in the ligand-containing electrolyte (Figure S11), suggesting additional energy input is required for the reaction in the presence of the ligand in the electrolyte.

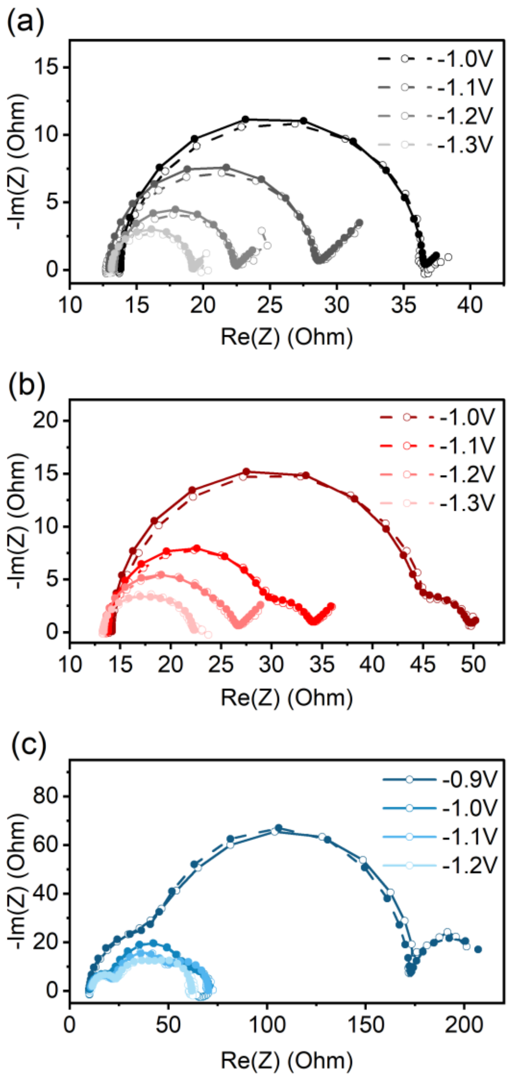

Figure S17. Potentiostatic electrochemical impedance spectra recorded for (a) pristine Cu, (b) layer-Cu in 0.1 M $KHCO_3$, and (c) layer-Cu in 1-Br_2-containing 0.1 M $KHCO_3$. All fit results are summarized in Table S2-4.

Potential / V vs RHE	R_{sol} / Ω	C_{dl} / μF·cm^{-2}	R_{ct} / Ω	W / Ω·s$^{-1/2}$
-1.0	13.8	29.1	22.6	0.8
-1.1	13.0	31.0	15.3	2.5
-1.2	13.3	27.4	8.9	1.7
-1.3	13.1	28.5	6.0	0.4

Table S2. Extrapolated parameters from the impedance spectra recorded on pristine Cu in 0.1 M $KHCO_3$ by fitting with equivalent circuit I in Figure S10.

Potential / V vs RHE	R_{sol} / Ω	C_{dl} / μF·cm^{-2}	R_{ct} / Ω	C_{film} / mF·cm^{-2}	R_{film} / Ω	W / Ω·s$^{-1/2}$
-1.0	14.2	22.0	30.7	6.0	4.7	-
-1.1	13.8	22.3	15.6	1.8	3.8	1.9
-1.2	13.4	23.0	10.8	0.7	2.1	2.0
-1.3	13.4	26.1	6.6	0.2	2.2	-

Table S3. Extrapolated parameters from the impedance spectra recorded on layer-functionalized Cu in 0.1 M $KHCO_3$ by fitting with equivalent circuit II in Figure S10.

Potential / V vs RHE	R_{sol} / Ω	C_{dl} / μF·cm^{-2}	R_{ct} / Ω	C_{film} / mF·cm^{-2}	R_{film} / Ω	C_L / μF·cm^{-2}	R_L / Ω
-0.9	10.1	14.2	117.8	18.32	42.5	2.5	45.0
-1.0	10.2	19.5	36.3	1.0	10.9	1.9	13.8
-1.1	10.6	20.8	29.0	0.5	17.2	1.0	13.0
-1.2	11.2	20.0	22.6	0.2	14.5	0.8	13.4

Table S4. Extrapolated parameters from the impedance spectra recorded on layer-functionalized Cu in ligand-containing 0.1 M $KHCO_3$ by fitting with equivalent circuit III in Figure S10.

18) *Quasi in situ* XPS spectra of layer-Cu after 3h of CO_2RR at -1.0 V_{RHE} in 0.1 M $KHCO_3$

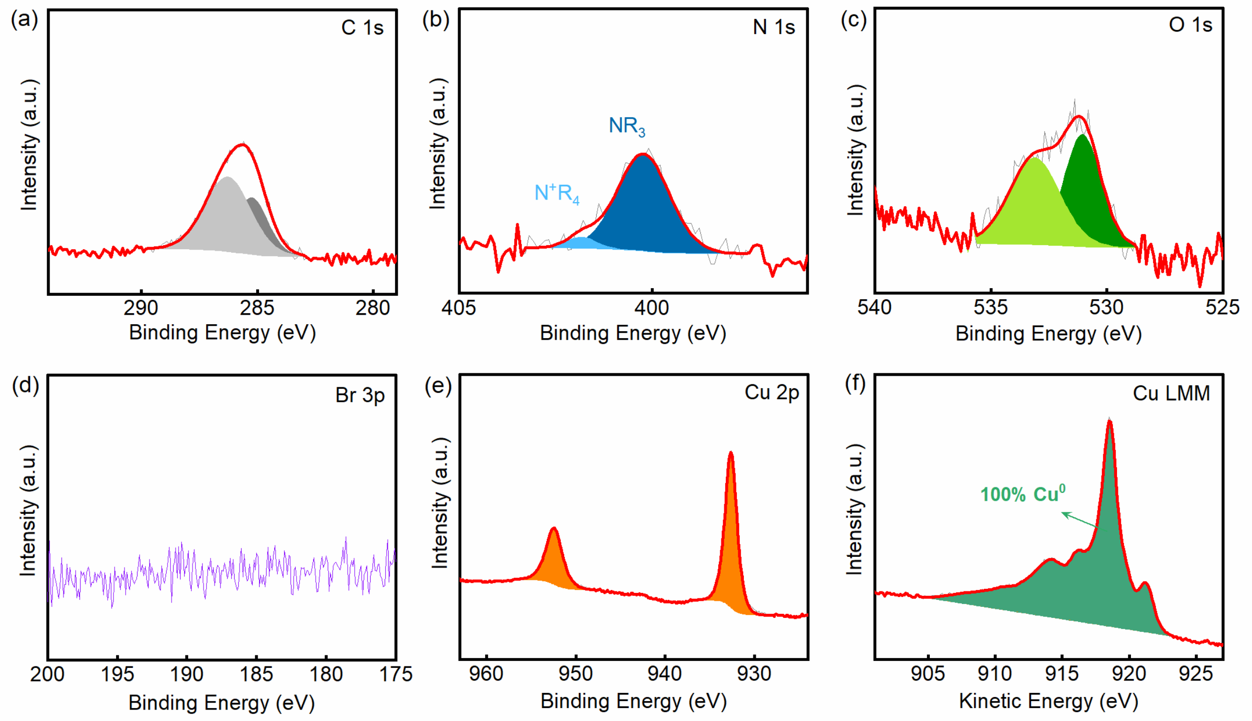

Figure S18. *Quasi in situ* XPS spectra of layer-Cu. (a) C 1s, (b) N 1s, (c) O 1s, (d) Br 3p, (e) Cu 2p, and (f) Cu LMM after 3h CO_2RR at -1.0 V_{RHE} in 0.1 M $KHCO_3$.

References

[1] Thevenon, A.; Rosas-Hernández, A.; Peters, J. C.; Agapie, T., *Angew. Chem. Int. Ed.* **2019,** *58* (47), 16952-16958.

[2] Shul, G.Weissmann, M.; Bélanger, D., *Langmuir* **2014,** *30* (22), 6612-6621.

[3] Horcas et al. *Rev. Sci. Instrum.* **2007**, *78*, 013705.
